# Reports on the Prevalence of Clinical Conditions Are More Convincing When Supported by Objective Evidence

**DOI:** 10.1371/journal.pntd.0000302

**Published:** 2008-09-24

**Authors:** Julius Schachter, Robin Bailey, Chandler R. Dawson, Thomas M. Lietman

**Affiliations:** 1 Department of Laboratory Medicine, University of California San Francisco, San Francisco, California, United States of America; 2 Department of Tropical Medicine, London School of Hygiene & Tropical Medicine, London, United Kingdom; 3 Proctor Foundation, University of California San Francisco, San Francisco, California, United States of America; Weill Medical College of Cornell University, United States of America

The accompanying research report by King and colleagues [1] describes a trachoma survey performed in Ayod County, southern Sudan. They performed a cross-sectional two-stage cluster survey of trachoma status in November 2006 and found levels of disease that rival those in the most hyper-endemic areas: 88% of children between ages 1 and 9 years had clinically active trachoma and the children were actually starting to show trichiasis (approximately 3%). In those over 14 years of age, 59% had clinically active trachoma, 14.6% had trichiasis, and 6.4% had corneal opacity. Trachoma was present in virtually all households; 98% had at least one person having active trachoma, and one-third of households had individuals with trichiasis.

Although a number of conditions generally considered to be risk factors for trachoma were commonly found (for example, only 5% of households had latrines and water availability was limited, with journey times to fetch water of more than 30 minutes), these alone could not account for the severity of the disease the authors described. What is certain is that in this extremely impoverished area of Sudan, blinding trachoma is a major public health problem. There has been a major collapse of infrastructure, and rudimentary public health measures have been lacking for many years.

However, when faced with reports of such high levels of disease, one must be highly sensitive to the possibility of systematic bias in the clinical grading. The World Health Organization scheme used here has proven invaluable for trachoma programs to assess which areas will require mass (community-wide) treatment. However, the grading scale is subjective, imprecise, and not a particularly good indicator for infection with the ocular strains of chlamydia that cause trachoma. It was developed for, and is best used within programs, but does not have the fine distinctions in grading clinical disease that one would like to see within scientific reports. King et al. report data from within a trachoma elimination program. They have undertaken evaluations and statistical analyses to ensure that the clinical scoring generated by their field workers was consistent and agreed with a standardized set of photos. However, these field workers were relatively inexperienced at dealing with trachoma and were specially trained for this program. The data they have generated will be valuable in the monitoring and evaluation of their trachoma elimination program.

There are good self-serving reasons for those performing surveys in the context of control programs to provide the most solid evidence of the validity of their baseline findings. Systematic overscoring of clinical disease at baseline will almost certainly result in a successful outcome for any intervention; it could make the results of ineffective programs appear good, and the results of good programs appear spectacular. It is far better to provide evidence for the baseline levels that either cannot be questioned, or which can be validated. Thus, the unbiased reader would really like to have something besides subjective clinical classifications.

A relatively easy way to document the validity of the clinical classifications is to take photographs of the conjunctivae and make these available for external review to ensure that there is no over-grading. Digital photography now offers the ability to generate high-quality records of the initial clinical findings for validation purposes. It is not clear to us why photography is not a routine tool used by programs to monitor their success. A further option would be to document *Chlamydia trachomatis* infection using current generation nucleic acid amplification tests (NAATs) to detect the causative agent of trachoma. While this may not be the current state-of-the-art for trachoma surveys, and has cost implications, it has proved a useful tool for documenting prevalence rates of chlamydial infection in trachoma-endemic areas, and the positivity prior to treatment correlates well with disease intensity [2]. To reduce costs, a sampling strategy could be developed to use NAAT testing before the program is implemented to estimate a baseline prevalence of infection, and to compare this to results obtained after implementation of the AFE aspects of the Surgery, Antibiotics, Facial hygiene and Environmental improvements (SAFE) strategy [3]. The lower prevalence of infection seen after treatment allows specimen pooling strategies to further reduce costs of NAAT testing. Infection is a more sensitive indicator of the success of antibiotic treatment than is clinical disease, which takes a longer time to resolve. Perhaps resources for NAAT testing were not available, and there were concerns about the use of swabs, and transport of specimens to the laboratory; but certainly in the era of digital photography it is not unreasonable to request documentation of clinical findings for such a report.

Having raised this caveat, we must congratulate King and colleagues for performing this survey under extraordinarily difficult conditions. They are working in an area where supplies are difficult to obtain, and where there is civil unrest. They have documented an extremely high prevalence of clinically active trachoma and blinding and pre-blinding lesions that indicate that attention will have to be paid to the trachoma problem in this area for decades to come, as the lesions progress. Certainly, there are other trachoma-endemic areas that are impoverished and where infrastructure collapse will have created an environment that allows trachoma to flourish to levels similar to those seen here. Indeed, studies in the past several years in Ethiopia have also found alarming rates of active disease and trichiasis, indicating an unprecedented need for corrective lid surgery. The work of King and colleagues will bring deserved attention to this problem.
